# Whole-body muscle MRI of patients with MATR3-associated distal myopathy reveals a distinct pattern of muscular involvement and highlights the value of whole-body examination

**DOI:** 10.1007/s00415-020-09862-9

**Published:** 2020-05-02

**Authors:** Alexander Mensch, Torsten Kraya, Felicitas Koester, Tobias Müller, Dietrich Stoevesandt, Stephan Zierz

**Affiliations:** 1 Department of Neurology, Martin-Luther-University of Halle-Wittenberg, Halle (Saale), Germany; 2 Department of Neurology, Klinikum St. Georg, Leipzig, Germany; 3 Department of Radiology, Martin-Luther-University of Halle-Wittenberg, Halle (Saale), Germany

**Keywords:** Matrin-3, MATR3, Distal myopathy, Whole-body MRI, Muscle MRI

## Abstract

**Objective:**

MATR3-associated distal myopathy is a rare distal myopathy predominantly affecting lower legs as well as wrist- and finger extensors. Whilst most distal myopathies are clinically and genetically well characterized, diagnosis often remains challenging. Pattern-based magnetic resonance imaging (MRI) approaches offer valuable additional information. However, a consistent pattern of muscular affection is missing for most distal myopathies. Thus, the aim of the present study was to establish a disease-specific pattern of muscular involvement in MATR3-associated distal myopathy using whole-body MRI.

**Methods:**

15 patients (25–79 years of age, 7 female) with MATR3-associated distal myopathy were subjected to whole-body MRI. The grade of fatty involution for individual muscles was determined using Fischer-Grading. Results were compared to established MRI-patterns of other distal myopathies.

**Results:**

There was a predominant affection of the distal lower extremities. Lower legs showed a severe fatty infiltration, prominently affecting gastrocnemius and soleus muscle. In thighs, a preferential involvement of semimembranous and biceps femoris muscle was observed. Severe affection of gluteus minimus muscle as well as axial musculature, mainly affecting the thoracic segments, was seen. A sufficient discrimination to other forms of distal myopathy based solely on MRI-findings of the lower extremities was not possible. However, the inclusion of additional body parts seemed to yield specificity.

**Interpretation:**

Muscle MRI of patients with MATR3-associated distal myopathy revealed a distinct pattern of muscular involvement. The usage of whole-body muscle MRI provided valuable additional findings as compared to regular MRI of the lower extremities to improve distinction from other disease entities.

**Electronic supplementary material:**

The online version of this article (10.1007/s00415-020-09862-9) contains supplementary material, which is available to authorized users.

## Introduction

Distal myopathies are a heterogeneous group of rare muscular disorders that vary in disease onset, mode of inheritance and clinical presentation [1]. Recent advances in genetics have broadened the genotypic spectrum of distal myopathies enormously. To date, nearly 20 distinct disease entities have been identified as classical distal myopathies. Moreover, a variety of muscular disorders show a facultative distal phenotype. Thus, the identification of the correct genotype as well as the evaluation of genetic variants of uncertain significance remain challenging and require a thorough clinical workup [2].

Imaging of the musculature offers valuable additional information to facilitate the diagnostic process as specific patterns of muscular involvement—with some muscles being selectively affected whilst others are spared—have been observed in different muscle diseases [3]. There are various studies that have aimed to establish specific patterns of muscular affection within different distal myopathies (e.g. [4–27].). However, the majority of reports rely on a relatively small number of individuals studied. Furthermore, most studies are lacking a systematic quantitative and qualitative assessment of the individual muscles (by means of a grading system) as well as information regarding inter-individual variability. In addition, selective imaging of the lower extremities instead of whole-body examination was used for pattern definition in most cases. Consistently, pattern-based approaches to identify the underlying genotype of patients with distal myopathy have yielded low sensitivity and specificity [28]. Hence, a structured evaluation of preferably large cohorts is needed to refine the individual patterns of muscular involvement and to improve the predictive value of information gained by muscle imaging.

Matrin-3-associated distal myopathy is a rare late-onset distal myopathy predominantly affecting the lower legs as well as finger and wrist extensors [29]. Dysphagia, dysphonia and a restrictive ventilation disorder are observed frequently throughout disease progression [30]. Since its first description as Vocal cord and pharyngeal weakness with distal myopathy (VCPDM) in 1998 and subsequent identification of the causative mutation in the MATR3-Gene (p.S85C, c.254C > G), a number of patients with Matrin-3-associatied distal myopathy have been identified. Hence, the clinical phenotype has been profoundly characterized [29, 31–35]. Although some of these reports also included muscle imaging, a comprehensive evaluation in terms of specific patterns, frequency of occurrence and severity of the observed muscular affection as well as dynamics throughout disease progression is missing [29, 31, 33, 35].

In this study, whole body-MRI scans of patients with genetically proven Matrin-3-associated distal myopathy were used with the aim to identify a specific pattern of muscular affection and to monitor the sequence of involvement. Furthermore, the obtained pattern was compared to findings in other distal myopathies to address the diagnostic value of the identified changes.

## Patients and methods

### Patients

Patients with genetically confirmed Matrin-3-associated distal myopathy (p.S85C/c.254C > G mutation) who underwent whole-body MRI in the course of routine diagnostic work-up were asked for permission to re-evaluate the obtained data for the present study. 15 patients (25–79 years of age, 7 female) out of 8 families were included in the current study. The majority of the patients (9 patients) were in mid-stage of the disease (41–55 years of age, disease duration 5–29 years), whilst each three were classified to be in disease onset (25–30 years of age, presymptomatic or myalgia only) and end-stage (67–79 years of age, disease duration 36–39 years, disease duration not known in one case) disease, respectively. While 4 of the patients (patients 4, 5, 10, 11) were previously described [29], 4 newly identified unrelated pedigrees were included in the current study. Table 1 summarizes the main characteristics of the patients.

**Table 1:** Included patients with MATR3-myopathy

Patient no.	1	2	3	4	5	6	7	8	9	10	11	12	13	14	15
Family	I	I	I	II	III	IV	V	I	I	VI	VII	I	VIII	I	I
Age															
* At disease onset*	pre	pre	pre	36	37	20	30	35	40	25	35	40	n.a	40	40
* At MR examination*	25	29	30	41	45	45	48	53	54	54	54	55	67	76	79
Disease duration at MR examination	–	–	–	5	8	25	18	18	14	29	19	15	–	36	39
BMI	27.7	27.8	23.7	31.1	24.2	20.8	30.1	25.9	29.8	26.1	44.2	25.1	23.8	22.9	29.6

*No.* number, *pre* presymptomatic, *BMI* body mass index

### MRI protocol and evaluation

Whole-body-Imaging was done on a 3.0 T MRI-system (Skyra; Siemens, Erlangen, Germany) using two flexible 18-channel transmit/receive surface body coils for neck, thorax, arms, abdomen and pelvis. A 36-channel angiography coil was used for the legs. The patient was placed in the supine position. Imaging included a T1-Weighted Dixon Turbo Spin Echo sequence in an axial plain and 8 mm slice thickness. The total amount of time required for the MRI examination was 45 min. No sedation or contrast agent was used.

The grade of affection of the individual muscle was determined using the five-point semi-quantitative grading scale established by Fischer et al. [10]. Briefly, stage 0 refers to a normal appearance, while decreased T1-signal intensity to a variable extent is found in stages 1–4 (1—slight, non-confluencing; 2—beginning confluence in less than 50% of the muscle; 3—confluence in more than 50% of the muscle; 4—replacement of the entire muscle).

Determination was conducted by an experienced radiologist (DS) who was blinded to the clinical data. A complete summary showing the individual patients’ determined Fischer-grades can be found in Suppl. Table 1.

### MATR3 MRI-pattern definition

To establish a comprehensive MRI-pattern of muscular affection, patients were first stratified in regard to disease duration (disease onset, mid-stage and end-stage disease). For pattern definition, only advanced disease stages (e.g. mid- and end-stage) were used. For each individual muscle, average as well as median Fischer grade and standard deviation was determined. Using these parameters, the grade of affection was classified into four categories—not/rarely affected (average Fischer grade 0–1.4, median Fischer grade 0), mild/variable affected (average Fischer grade 1.5–2.4, median Fischer grade 1–2, standard deviation > 1.5), moderately affected (average Fischer grade 2.5–3.4, median Fischer grade 3) and early/severely affected (average Fischer grade ≥ 3.5, median Fischer grade 4).

### Pattern evaluation

To test the reliability of the obtained MRI-pattern, all patients with MATR3-myopathy included in this study were compared to the MRI-pattern of muscular affection identified in this study. The derived MATR3 MRI-pattern was dichotomized, only distinguishing between unaffected (not or mild/variable affected) and affected (moderately and early/severely affected) muscles. In patients, all muscles with a Fischer grade > 1 were considered to be affected. For each patient, the mismatch to the defined MATR3-pattern was determined. In this regard, a mismatch was defined as either a muscle that was affected in the individual patient but not in the defined MATR3-pattern or vice versa a muscle that appeared to be not affected in the individual patient but was defined affected in the MATR3-pattern. A mismatch ≤ 20% of all muscles studied in the individual patient was supposed a good congruency to the MATR3-pattern. Hence, the portion of patients fulfilling this criterion was identified.

### Comparison of MATR3 MRI-pattern to other distal myopathies

Patterns of muscular involvement for the individual distal myopathies were defined on the basis of previous findings in distal myopathies. The work of Bugiardini et al*.* served as the basis for pattern definition [28]. Furthermore, a selective literature review was done using the terms “MRI”, “magnetic resonance imaging”, “MR” and “imaging” in connection with “distal myopathy” as well as the respective disease entities. A comprehensive list of the literature used for pattern definition can be found in Suppl. Table 2. In addition to the known “classical” distal myopathies (obligatory distal phenotype), several myopathies with comparably high prevalence and potential predominant distal affection were included (Facioscapulohumeral muscular dystrophy 1, Myotonic dystrophy 1, sporadic Inclusion body myositis, Hereditary myopathy with early respiratory failure).

The established literature-based pattern was then compared to MATR3-pattern and mismatch was determined. Again, a mismatch ≤ 20% was defined as relevant congruency.

## Results

### Muscular affection in MATR3-myopathy

**Fig. 1:**
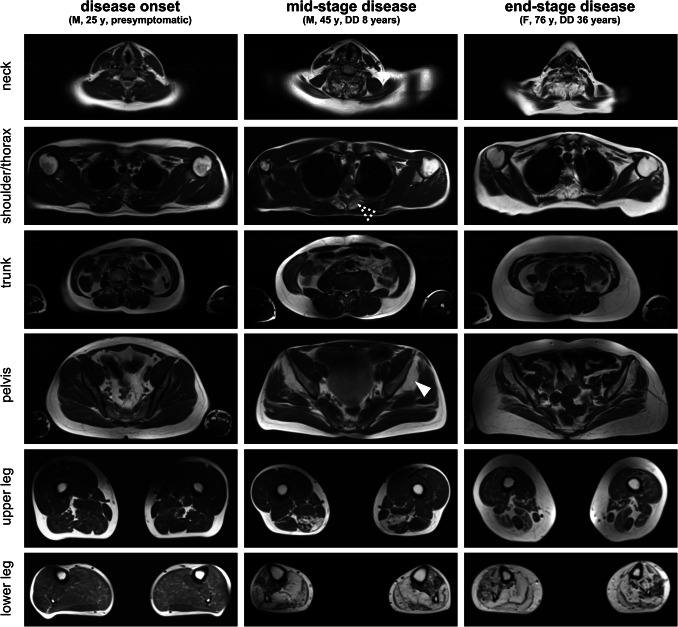
Magnetic resonance imaging in MATR3-associated distal myopathy. Representative T1-weighted MRI slices of head/neck, shoulder/thorax, trunk, pelvis, upper and lower leg in patients with different disease stages of MATR3-associated distal myopathy. Note the early affection of gluteus minimus muscle (white arrowhead) and thoracic segments of paraspinal musculature (dashed arrowhead) while gluteus medius and maximus muscles as well as cervical and lumbar segments of paraspinal musculature are relatively spared. *DD* disease duration, *y* years, *F* female, *M* male

**Fig. 2:**
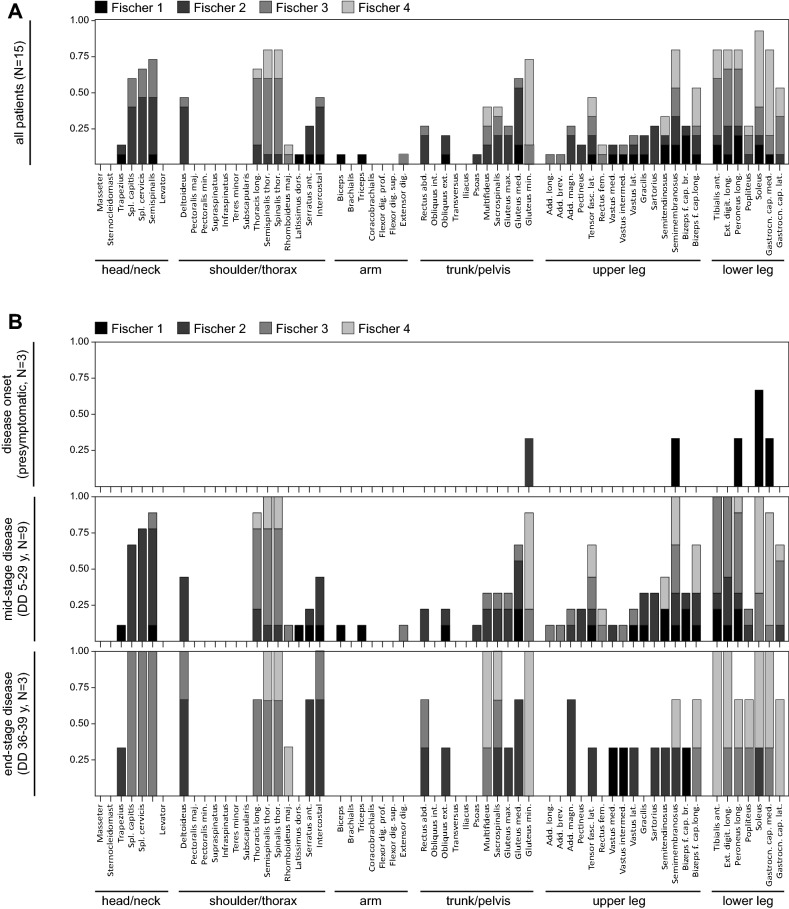
Grade of affection for individual muscles as determined by Fischer grading. The proportion of respective Fischer grades (Fisher 1–Fischer 4) observed in individual muscles is shown for all patients (**a**) as well as disease onset, mid- and end-stage disease (**b**). *DD* disease duration, *y* years, *Spl.* Splenius, *maj.* major, *min.* minor, *long.* longus, *thor.* thoracis, *dors.* dorsi, *ant.* anterior, *prof.* profundus, *sup.* superficialis, *dig.* digitorum, *abd.* abdominis, *int.* internus, *ext.* externus, *max.* maximus, *med.* medius, *min.* minumus, *brev.* brevis, *magn.* magnus, *fasc. lat.* fasciae latae, *fem.* femoris, *med.* medialis, *intermed.* intermedius, *lat.* lateralis, *cap. brev.* caput breve, *cap. long.* caput longum, *cap. med.* caput mediale, *cap. lat.* caput laterale

Muscular involvement was mainly symmetrical. A predominant affection of the lower extremities with an emphasis on the lower legs was observed. In lower legs, a predominant involution of soleus muscle as well as medial head of gastrocnemius muscle was detected. However, the anterior and lateral compartment (e.g. tibialis anterior, extensor digitorum longus and peroneus longus muscle) were also involved in most of the cases, though to a lesser extent. A variable degeneration of lateral head of gastrocnemius muscle was seen. However, the deep posterior compartment was mainly unaffected. In thighs, a selective affection of parts of the hamstring muscles (semimembranosus muscle and the long head of the biceps femoris muscle) was observed whereas the anterior and medial compartments were mostly spared. Imaging of the trunk showed early and severe involvement of the gluteus minimus muscle. In contrast, gluteus medius muscle showed a variable grade of affection while gluteus maximus muscle appeared to be not relevantly involved. Evaluation of the paraspinal musculature revealed a scattered degeneration predominantly affecting thoracic parts of the axial musculature (e.g. thoracis longus, semispinalis thoracis and spinalis thoracis muscles). The cervical and lumbar parts of paraspinal musculature appeared only mildly affected. Except for the deltoid muscle, that—especially in the ventral part—showed a moderate involution, no relevant involvement of the musculature of shoulder and arm was seen. Representative examples of whole body imaging findings in MATR3-myopathy are depicted in Fig. 1 while the observed grade of alteration for the individual muscles as determined by Fischer grading are shown in Fig. 2.

### Dynamics throughout disease progression

At disease onset, there was already a slight (Fischer Grade 1 and 2) involvement in muscles that appeared to be severely affected in advanced disease stages. Especially, gluteus medius muscle as well as the lower limb muscles (e.g. soleus and peroneus longus muscles and the medial head of the gastrocnemius) showed mild involution in some of the patients (Fig. 2b, upper panel). During the course of the disease, a contemporaneous degeneration of the typically affected muscles was observed (Fig. 2b, middle panel), while not or rarely affected muscles appeared to be spared up to late stages of the disease, showing only mild involution that could be ascribed to age-related changes (Fig. 2b, lower panel).

### Pattern definition and evaluation

**Fig. 3:**
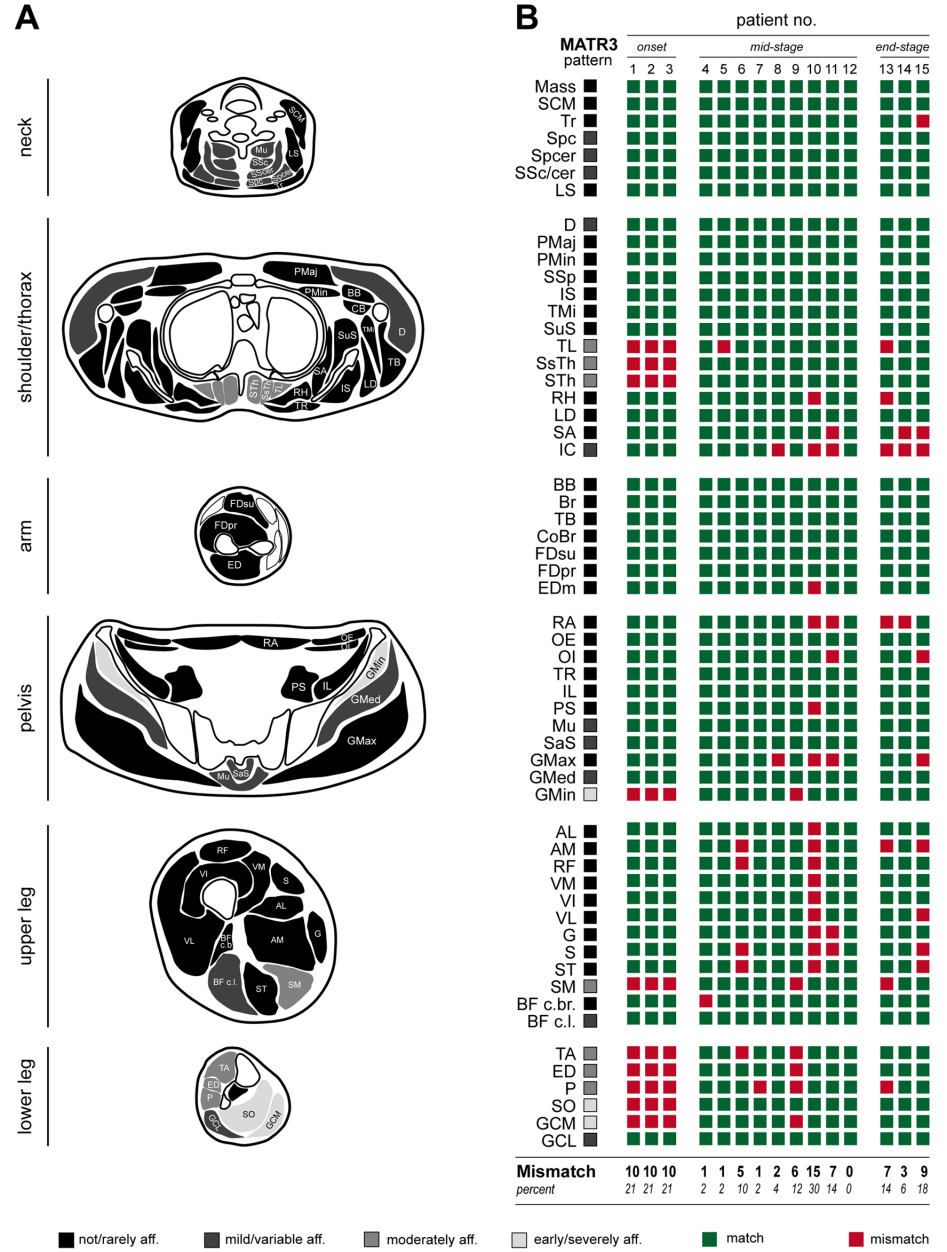
Established pattern of muscular affection in MATR3-associated distal myopaty. **a** Schematic representation of the established pattern of muscular affection in MATR3-associated distal myopathy, stratified to not/rarely, mild/variable, moderate and early/severely affected. **b** Evaluation of the obtained pattern in patients with MATR3-myopathy as shown by match/mismatch of the individual muscles to the established pattern. *Mass* masseter, *SCM* sternocleidomastoid, *Tr* trapezius, *Spc* splenius capitis, *Spcer* splenius cervicis, *SSc/cer* semispinalis capitis/cervicis, *LS* levator scapulae, *D* deltoideus, *Pmaj* pectoralis major, *Pmin* pectoralis minor, *SSp* supraspinatus, *IS* infraspinatus, *Tmi* teres minor, *SuS* subscapularis, *TL* thoracic longissimus, *SsTh* semispinalis thoracis, *STh* spinalis thoracis, *RH* rhomboideus major, *LD* latissimus dorsi, *SA* serratus anterior, *IC* intercostal, *BB* biceps brachii, *Br* brachialis, *TB* triceps brachii, *CoBr* coracobrachialis, *Fdsu* flexor digitorum superficialis, *FDpr* flexor digitorum profundus, *Edm* extensor digitorum manus, *RA* rectus abdominis, *OE* obliquus externus, *OI* obliquus internus, *TR* transversus abdominis, *IL* iliacus, *PS* psoas, *Mu* multifidus, *SaS* sacrospinalis, *GMax* gluteus maximus, *GMed* gluteus medius, *GMin* gluteus minimus, *AL* adductor longum, *AM* adductor magnus, *RF* rectus femoris, *VM* vastus medialis, *VI* vastus intermedius, *VL* vastus lateralis, *G* gracilis, *S* sartorius, *ST* semitendinosus, *SM* semimembranosus, *BF c.br.* biceps femoris short head (caput breve), *BF c.l.* biceps femoris long head (caput longum), *TA* tibialis anterior, *ED* extensor digitorum, *P* popliteus, *TP* tibialis posterior, *SO* soleus, *GCM* gastrocnemius medialis, *GCL* gastrocnemius lateralis, *aff.* affected

A comprehensive pattern of muscular involvement in MATR3-associated myopathy was defined on the basis of the Fischer grading system (for details see “Methods” section). Three muscles, namely soleus muscles, the medial head of gastrocnemius muscle and gluteus minimus muscle were identified as early/severely affected. Tibialis anterior, extensor digitorum, peroneus longus and semimembranosus muscles as well as the thoracic parts of paraspinal musculature were categorized as being moderately affected. A mild/variable involution was identified for the lateral head of gastrocnemius and biceps femoris muscles, gluteus medius muscle, deltoideus muscle and cervical/lumbar parts of paraspinal musculature. All other muscles were defined as been not or rarely affected. A comprehensive summary of the defined MATR3-pattern is given in Fig. 3a.

To evaluate the practicability of the defined MATR3-pattern, mismatches to the findings in the individual patients were identified (Fig. 3b, Table 2). For all patients, a median mismatch of 6 muscles (12% of all examined muscles) was determined. The best match to the defined pattern was observed in the group of individuals with mid-stage disease, showing a median mismatch of 2 muscles (4%). While patients in end-stage disease showed a rather good match to the defined MATR3 pattern (median mismatch 7 muscles), a consistently poor match was seen in the disease onset group (median mismatch 10 muscles).

**Table 2:** Mismatch between defined MATR3-pattern and findings in individual patients

	All patients	Onset	Mid-stage	End-stage
**Mismatch**				
Average * No of muscles with mismatch (percent of all muscles)*	5.8 (12%)	10 (21%)	4.2 (9%)	6.3 (13%)
Median * No of muscles with mismatch (percent of all muscles)*	6 (12%)	10 (21%)	2 (4%)	7 (14%)
Range *No of muscles with mismatch (percent of all muscles)*	0–15 (0–30%)	All 10 (21%)	0–15 (0–30%)	3–9 (6–18%)
**Mismatch < 20% (≤ 6 muscles)**				
* No of patients (percent)*	11/15 (73%)	0/3 (0%)	8/9 (89%)	3/3 (100%)
**Mismatch < 10% (≤ 3 muscles)**				
* No of patients (percent)*	6/15 (40%)	0/3 (0%)	5/9 (55%)	1/3 (33%)

Overall, in 11 out of 15 patients (73%) a mismatch less than 20% of all examined muscles was observed. None of the patients in the disease onset group showed a sufficient match with the MATR3-pattern. A sufficient congruency (defined as mismatch < 20%) was observed for 89% of the patients in mid-stage and 100% of the patients in end-stage disease. An even stricter match to the defined MATR3-pattern (mismatch < 10%) was observed in 5 out of 9 patients in mid-stage disease (55%), while only one specimen (33%) in late-stage disease fulfilled this criterion.

### Comparison to other distal myopathies

**Fig. 4:**
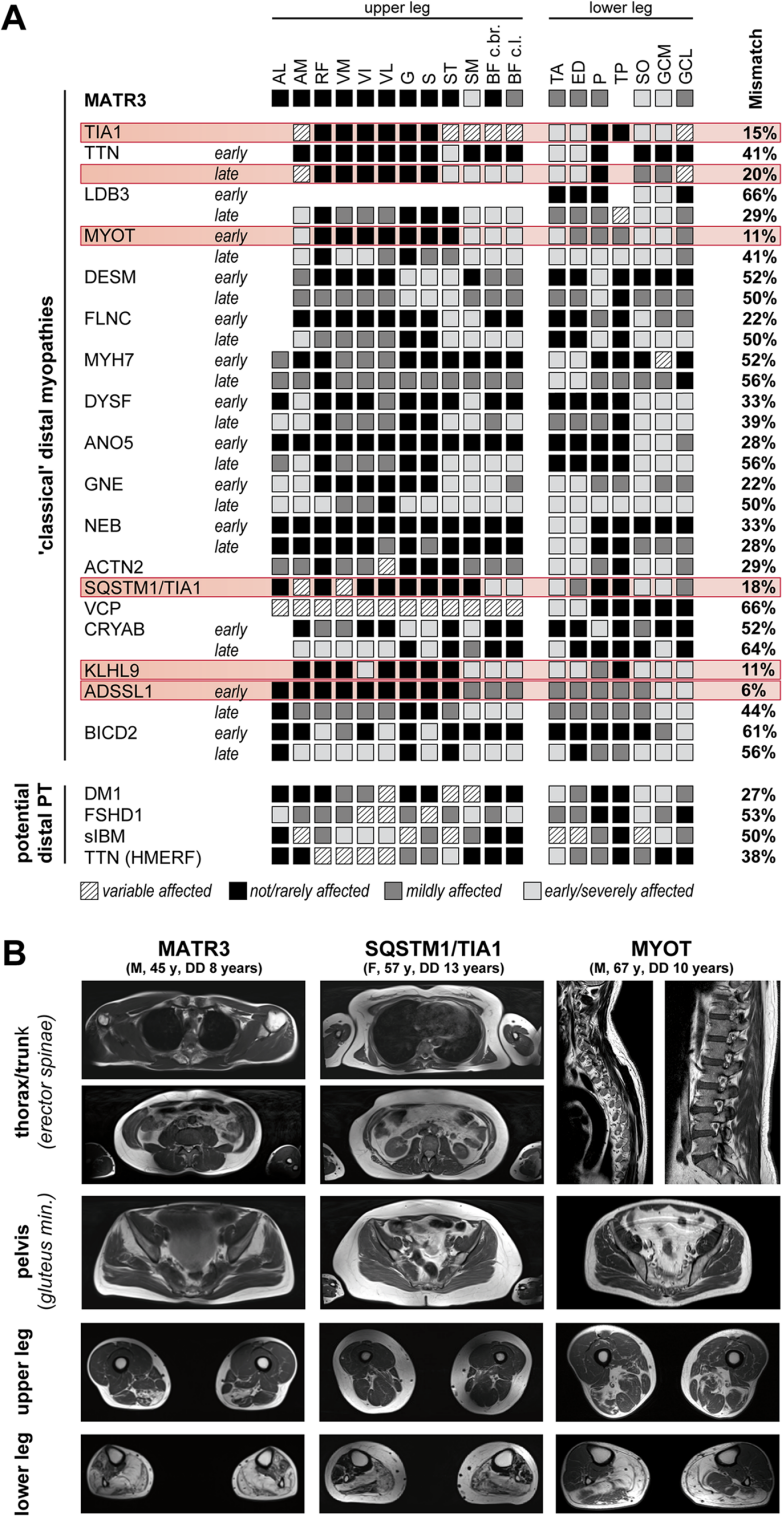
Comparison of the established MATR3-pattern to other distal myopathies. **a** Literature-based pattern of different distal myopathies and the respective mismatch to the established MATR3-pattern. Both ‘classical’ distal myopathies (obligatory distal phenotype), as well as myopathies with comparably high prevalence and potential predominant distal phenotype (potential distal PT) are included. Disease entities showing relevant similarities (mismatch < 20%) are highlighted in red. **b** Sample MRI findings in patients with distal myopathies determined to show similar patterns in lower limb muscular affection compared to MATR3-myopathy. Gluteal and paraspinal musculature is spared in patients with MYOT- and SQSTM1/TIA1-associated distal myopathy. *DD* disease duration, *y* years, *PT* phenotype, *F* female, *M* male, *FSHD* Facioscapulohumeral muscular dystrophy 1, *DM1* Myotonic dystrophy 1, *sIBM* sporadic Inclusion body myositis, *HMERF* Hereditary myopathy with early respiratory failure, *AL* adductor longum, *AM* adductor magnus, *RF* rectus femoris, *VM* vastus medialis, *VI* vastus intermedius, *VL* vastus lateralis, *G* gracilis, *S* sartorius, *ST* semitendinosus, *SM* semimembranosus, *BF c.br.* biceps femoris short head (caput breve), *BF c.l.* biceps femoris long head (caput longum), *TA* tibialis anterior, *ED* extensor digitorum, *P* popliteus, *TP* tibialis posterior, *SO* soleus, *GCM* gastrocnemius medialis, *GCL* gastrocnemius lateralis, *aff.* affected

To determine the diagnostic practicability of the established MATR3-pattern, it was compared to literature-based lower-limb MRI patterns of other distal myopathies (Fig. 4a). A relevant discrimination (e.g. mismatch > 20%) of the MATR3-pattern to the majority of distal myopathies (16 out of 22 disease entities) was seen. In contrast, relevant similarities were observed in 6 distal myopathies (TIA1, late-stage TTN, MYOT, SQSTM1/TIA1, KLHL9, ADSSL1). Comprehensive information regarding affected muscles other than lower limb muscles were lacking for most of these distal myopathies, as only a few underlying studies applied whole-body MRI (2 out of 15, Table 3). Based on the available literature, a frequent affection of gluteal muscles was observed in MYOT- associated distal myopathy. However, a selective involution of gluteus minimus muscle as in MATR3-associated myopathy was not described (Table 3). Muscular involution of the paraspinal musculature was again solely reported in MYOT-associated distal myopathy. While a selective involvement of the thoracic paraspinal segments has not been described, there has been no explicit discrimination between lumbar, thoracic and cervical segments in this study (Table 3). Furthermore, respective changes were not present in MR-studies from patients with MYOT- and SQSTM1/TIA1-associated distal myopathy overseen in the neuromuscular research centre in Halle (Fig. 4b).

**Table 3:** MRI studies in distal myopathies showing similar patterns of lower limb muscular affection as compared to MATR3-myop

Reference	No. of patients	Whole body MRI	Individual muscles	Scoring system	Gluteal musculature	Paraspinal musculature
**TIA1**						
*Mahjneh (2004)* [14]	11	N	Y	Y	n.d	n.d
*Ahlberg (1994)* [4]	6	N	Y	N	n.d	n.d
**TTN**						
*Mahjneh (2004)* [14]	22	N	Y	Y	n.d	n.d
**MYOT**						
*Berciano (2008)* [5]	8	N	Y	Y	All glutei involved (min. and med. > max.)	n.d
* McNeill (2009)* [15]	8	N	Y	Y	n.d	n.d
*Olive (2011)* [18]	17	N	Y	Y	n.d	n.d
*Fischer (2008)* [10]	12	N	Y	Y	All glutei involved (min. and med. > max.)	n.d
*Schramm (2008)* [25]	5	Y	Y	Y	Glutei involved, no discrimination	Erector spinae involved, no segmental discrimination
**SQSTM1/TIA1**						
*Niu (2018)* [16]	3	n.a	N	N	n.d	n.d
*Lee (2018)* [12]	9	N	N	N	Affected in 1 patient, no discrimination	Affected in 1 patient, no discrimination
*Bucelli (2015)* [6]	1	N	N	N	n.d	n.d
**KLHL9**						
*Cirak (2010)* [7]	1	N	N	N	n.d	n.d
**ADSSL1**						
*Park (2016)* [21]	2	N	N	N	Not affected	n.d
* Park (2017)* [22]	3	Y	N	N	Not affected	Not affected

*No.* number, *Y* yes, *N* no, *n.a.* not annotated, *n.d.* not determined

## Discussion

In this study, a comprehensive pattern of muscular involvement in MATR3-associated distal myopathy has been established. To our knowledge, this is the largest cohort of patients with MATR3-myopathy studied so far. Furthermore, whole-body MRI as well as a semi-quantitative, scoring-based evaluation system is used for the first time to evaluate muscular affection in MATR3-myopathy.

Overall, the observed muscular involution meets very well with the typical clinical aspects seen in MATR3-myopathy (e.g. reported in [29, 32]). Corresponding to the initial symptoms complaint by the patients, a predominant affection of the distal lower limbs was seen. However, in contrast to the initially present bilateral foot-drop, MRI-evaluation showed the posterior compartment to be involved in early disease stages. One explanation of this discrepancy might be the larger volume of the posterior compartment in comparison to the anterior compartment and thus higher capacity to compensate for the beginning involution. Furthermore, most of the patients clinically exhibit a relevant weakness of wrist-/finger extensors, whereas respective MRI-changes were not seen in the present study. The low local resolution as well as the forearms being placed at the edge of the field of view and thus being particularly susceptible to artefacts may account for this phenomenon. Additional sequences focussing on the distal upper limbs or thin, flat muscles like trapezius muscle (e.g. coronal and sagittal sections) may further yield the diagnostic value but have to be carefully pondered against the increasing acquisition time (45 min with the protocol used in this study). The observed selective involvement of the thoracic segments of paraspinal musculature corresponds very well to the camptocormia frequently observed in advanced disease stages of MATR3 myopathy (unpublished observation).

Based on the grading system applied, a comprehensive pattern of muscular affection in MATR3-associated myopathy could be established, that showed a feasible match to the individual patients. However, validation of the MATR3-pattern was achieved solely on the basis of the patients that also served for pattern definition. This obvious limitation of the study mainly relates to the low prevalence of the disease and thus lack of additional individuals for thorough validation. In this regard, the only available publication addressing whole-body MRI in MATR3-associated distal myopathy apart from the present study is a case report describing one french patient [31]. The pattern of muscular involvement reported by Barp et al. fits well with the changes observed in the present study, although explicit information regarding individual muscle involvement is missing. Nonetheless, additional prospective studies including newly identified individuals with MATR3-myopathy are needed to further address this issue.

Overall, there was a good congruency of the MATR3-pattern with the individual muscular affection observed in patients. However, some patients (in particular patient 10 and 11) showed a relevant mismatch. Patient 10 had been subjected to high doses of glucocorticoids (non-related to muscular disease) for several years that might have led to additional muscular involution by means of a corticosteroid-induced myopathy. The discrepancies observed in this patient mainly apply to the proximal muscles of the lower extremity, which are predominantly affected in corticosteroid-induced myopathy [36, 37]. In Patient 11, the mismatch might at least in parts rely on excessive overweight (body mass index 44 kg/m^2^) leading to diffuse fatty infiltration of the musculature [38, 39]. In this regard, quantitative MRI assessment methods would be more feasible allowing normalisation to body mass index or mean fat fraction, respectively.

Noteworthy, MATR3-patients at disease onset (presymptomatic or myalgia only) did not match with the MATR3-pattern. Thus, the established pattern may only be useful in patients that show clinical weakness and not be suitable for early identification of oligosymptomatic patients. However, even patients that were imaged in a comparably short interval to symptom onset (patients 4 and 5, disease duration 5 and 8 years, respectively) displayed a good congruency to the defined pattern (mismatch 2% each). This suggests that the predominantly affected muscles are consistently involved at an early timepoint in disease progression.

When comparing the MATR3-pattern to literature-based patterns of other distal myopathies, a good distinction to most of the disease entities was seen. However, a relevant number of studies on disease-specific patterns were only based on a small sample size or did not apply semi-quantitative scoring systems. Furthermore, in most cases an explicit annotation regarding the individual grade of affection of all studied muscles is missing. Thus, the literature-based patterns used in this study might not fully account for the alterations to be expected in some disease entities. In this context, the missing validation of the defined patterns beyond literature evidence appears to be another limitation of the approach used in this study.

Interestingly, none of the myopathies with potential distal phenotype showed a relevant congruency to MATR3-myopathy. This might partly be due to the relevant affection of proximal muscle groups reported in all available MRI-studies concerning these disease entities. Reports addressing MRI-finding in patients with exclusive predominant distal affection are missing (see Suppl. Table 2 for details).

Despite the satisfying delineation from most distal myopathies using the findings of the lower limb alone, a relevant similarity was found for six distal myopathies. Hence, a sufficient discrimination on the basis of a sole examination of the lower limbs might not be possible.

Studies using whole-body MRI are missing for most of the distal myopathies with relevant congruency to MATR3-associated myopathy. On the basis of the available literature, patterns of gluteal and paraspinal affection found in MYOT-associated distal myopathy were divergent to the findings in MATR3-myopathy. This included also a patient with MYOT-associated distal myopathy overseen in the neuromuscular research centre in Halle. Furthermore, the only available study using whole-body MRI in MYOT-myopathy has reported frequent involvement of rhomboidei muscles as well as variable affection of proximal muscles of the upper extremities [25]. These findings were not observed in MATR3-myopathy and thus may further facilitate discrimination of both myopathies. Additionally, gluteal and paraspinal changes as observed in MATR3-myopathy were not seen in whole-body examination of a patient with SQSTM1/TIA1-associated distal myopathy, a distal myopathy that displays similar lower limb findings as compared to MATR3-associated myopathy.

Under this aspect, the usage of whole-body imaging offers additional information regarding sufficient delineation of different disease entities. Thus, the findings of the present study further support the recently published recommendations of the MYO-MRI consortium, emphasizing the role of whole-body MRI in diagnosing and monitoring neuromuscular disorders [40]*.* Whether the defined categories of muscular involvement, the used parameters and set margins are applicable for other studies using different disease entities remains elusive.

The approach presented in this study appears to be considerably operational. Integrational use of—among others—clinical presentation, family history and muscle biopsy findings along with MRI-examination should further facilitate the accuracy of discrimination between different distal myopathies. For instance, KLHL9- and ADSSL1-associated distal myopathies are reported to have a rather early onset between 10 and 20 years of age, while first symptoms in MYOT-associated distal myopathy are occurring between 50 and 60 years of age [7, 21, 22, 41, 42]. Thus, they might be easily distinguished from MATR3-myopathy that usually shows an onset between 30 and 40 years of age [29]. MYOT-myopathy usually presents with a myofibrillar histopathology, that has not been identified in MATR3-associated distal myopathy [43]. While TIA1- and SQSTM1/TIA1-associated distal myopathy display a rather identical clinical phenotype compared to MATR3 (in terms of onset, muscle weakness and histopathology), none of these disease entities typically include a relevant dysphagia and dysphonia [12, 16, 44, 45]. Autosomal dominant TTN-associated myopathy (Udd myopathy) shows no upper limb involvement and is usually restricted to the anterior compartment of the lower legs, while the posterior compartment is only mildly affected in late disease stages [14, 46]. Thus, a sufficient delineation to MATR3-associated myopathy appears feasible.

In summary, the distinct pattern of muscular affection established in this study expands the diagnostic opportunities in MATR3-associated distal myopathy in terms of discrimination to other distal myopathies. It emphasizes the benefits of semi-quantitative, scoring-based approaches and explicit delineation of all studied muscles. Furthermore, the data presented highlights the potential of whole-body examination for further delineation of different disease entities. In this respect, the conclusions drawn from this study do not only apply for distal myopathies but for MRI-based studies in neuromuscular disorders in general.

## Availability of data and material

All data used for this study is available in deidentified form in the Electronic Supplementary Material section.

## Electronic supplementary material

Below is the link to the electronic supplementary material.

Supplementary file 1 (XLSX 24 kb)

Supplementary file 2 (XLSX 13 kb)
